# Clinicopathological and Prognostic Comparison of Primary Pulmonary Adenoid Cystic Carcinoma and Mucoepidermoid Carcinoma: A Single-Center Study

**DOI:** 10.3390/jcm15187194

**Published:** 2026-09-16

**Authors:** Neslihan Akanıl Fener, Nurcan Ünver, İbrahim Aras, Melike Ülker

**Affiliations:** 1 Department of Pathology, Yedikule Chest Diseases and Thoracic Surgery Training and Research Hospital, Health Sciences University, 34668 Istanbul, Turkey; 2 Department of Thoracic Surgery, LIV Hospital, Istinye University Faculty of Medicine, 34340 Istanbul, Türkiye

**Keywords:** primary pulmonary salivary gland-type tumors, adenoid cystic carcinoma, mucoepidermoid carcinoma, disease-free survival

## Abstract

**Objectives:** Primary pulmonary salivary gland-type tumors are rare neoplasms, with adenoid cystic carcinoma (ACC) and mucoepidermoid carcinoma (MEC) being the most common histological subtypes. This study aimed to compare the clinicopathological characteristics, treatment outcomes, and survival of patients with ACC and MEC. **Methods:** We retrospectively analyzed 59 patients diagnosed with primary pulmonary salivary gland-type tumors between 2016 and 2025. The cohort included 37 patients with ACC and 22 with MEC. Demographic, clinicopathological, treatment-related, and survival data were compared between the two groups. **Results:** ACC was significantly associated with tracheal localization (62.2% vs. 13.6%, *p* < 0.001), positive surgical margins (35.1% vs. 9.1%, *p* = 0.019), and a higher recurrence/metastasis rate (32.4% vs. 4.5%, *p* = 0.020). Overall survival did not differ significantly between ACC and MEC (*p* = 0.530), whereas disease-free survival was significantly lower in the ACC group (*p* = 0.009). Positive surgical margins were associated with inferior disease-free survival (*p* = 0.036). Among ACC patients, a predominantly solid histopathological pattern was significantly associated with worse overall survival (*p* = 0.034). **Conclusions:** Although ACC and MEC are traditionally grouped as pulmonary salivary gland-type tumors, they demonstrate distinct clinicopathological and prognostic characteristics. ACC is associated with greater surgical complexity, higher recurrence risk, and poorer disease-free survival, emphasizing the importance of achieving negative surgical margins and maintaining long-term follow-up.

## 1. Introduction

Primary pulmonary salivary gland-type tumors constitute a rare group of neoplasms that are thought to arise from the submucosal glands of the tracheobronchial tree and account for only a very small proportion of all primary lung tumors. Histologically, these tumors show morphological similarities to their counterparts arising in the major and minor salivary glands. Adenoid cystic carcinoma (ACC) and mucoepidermoid carcinoma (MEC) are the most common histologic subtypes within this group [1,2].

Two main perspectives have emerged in the literature regarding the clinical behavior of these tumors. According to one view, primary pulmonary salivary gland-type tumors are generally slow-growing neoplasms with a more favorable prognosis than conventional primary lung malignancies, and long-term survival can be achieved with surgical treatment. This interpretation is supported particularly by studies reporting limited biological aggressiveness and favorable survival outcomes in low-grade MEC cases [3,4].

In contrast, a second view suggests that primary pulmonary salivary gland-type tumors should not be regarded as a single homogeneous group of indolent neoplasms. According to this perspective, ACC and MEC may differ in terms of anatomical distribution, growth pattern, surgical treatment requirements, risk of recurrence or metastasis, and survival outcomes. Although ACC is generally considered a slow-growing tumor, it is regarded as a more challenging subtype during long-term follow-up because of its tendency for central airway and tracheal involvement, submucosal and infiltrative spread, positive surgical margins, local recurrence, and late distant metastasis [1,2,5]. Therefore, ACC and MEC may need to be evaluated not merely as tumors within the same spectrum, but as two distinct subgroups with different clinicopathological risk profiles.

Current evidence on these tumors is derived mainly from case reports, small single-center series, systematic reviews, and large database analyses. Although large database studies are valuable because of their sample size, they often provide limited information on detailed pathological variables such as surgical margin status, microscopic residual disease, perineural invasion, lymphovascular invasion, necrosis, spread through air spaces (STAS), pleural invasion, dominant histopathological pattern, and recurrence or metastasis patterns [1,2,6]. Conversely, single-center series may allow more detailed pathological assessment, but their limited sample size may restrict meaningful comparisons between subtypes and survival analyses. Accordingly, it remains unclear which clinicopathological features distinguish primary pulmonary ACC from MEC and how these differences are reflected in recurrence, metastasis, and survival outcomes. In this context, the study sought to address whether ACC and MEC truly demonstrate similar indolent behavior or should instead be considered two distinct subgroups with different clinicopathological risk profiles.

## 2. Materials and Methods

This retrospective study was designed to compare the clinical, radiological, histopathological, treatment-related, and prognostic characteristics of patients diagnosed with primary pulmonary adenoid cystic carcinoma (ACC) and mucoepidermoid carcinoma (MEC). A total of 59 patients diagnosed with primary pulmonary salivary gland-type tumors at Yedikule Chest Diseases and Thoracic Surgery Training and Research Hospital between 2016 and 2025 were included in the study.

**Figure 1:**
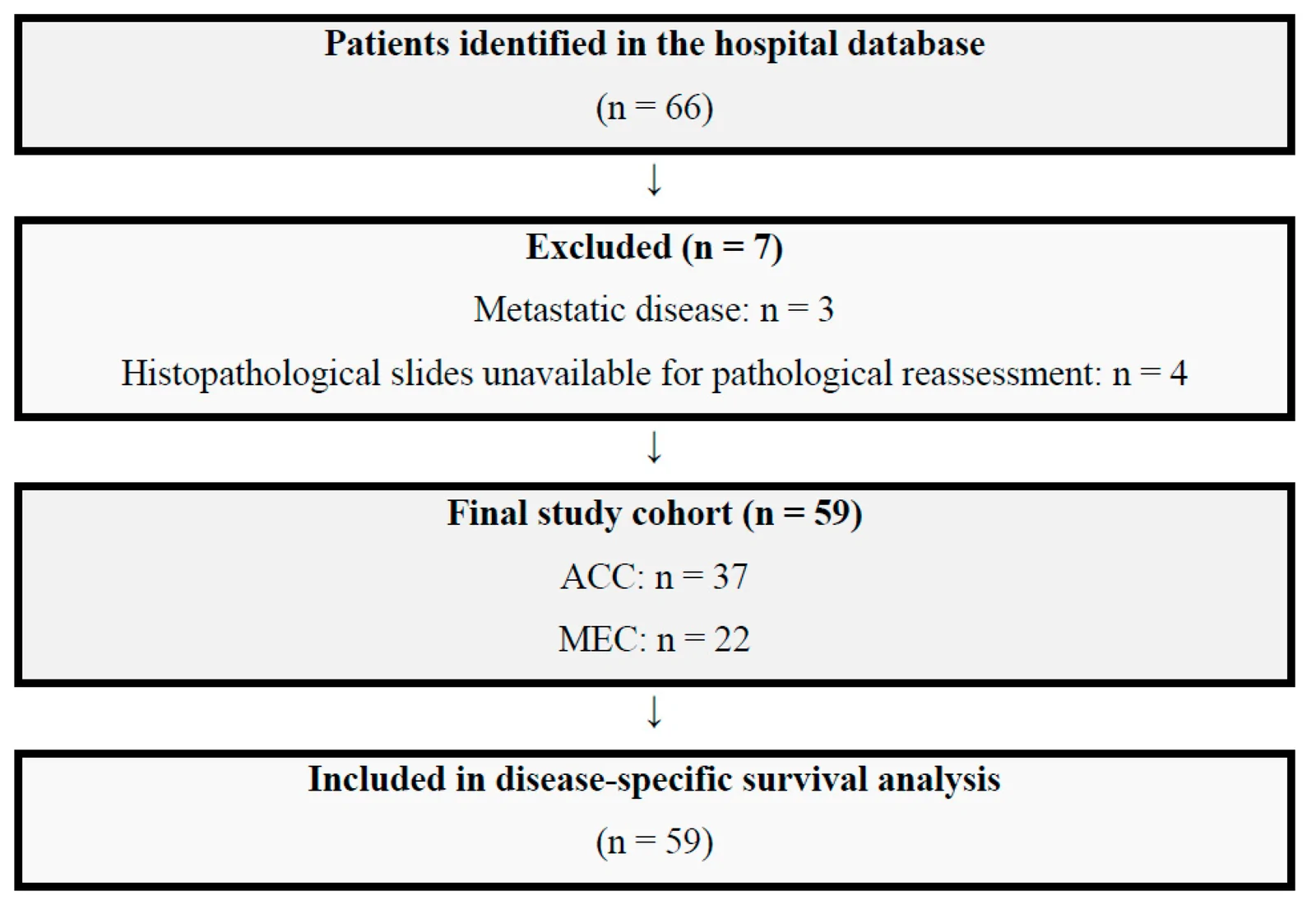
The study flowchart.

The study cohort consisted of 37 patients with histopathologically confirmed ACC and 22 patients with histopathologically confirmed MEC. Patients were eligible for inclusion if they had a histopathologically confirmed salivary gland-type tumor arising in the lung, trachea, or tracheobronchial tree and had available clinical follow-up data. The study flowchart has been presented in Figure 1. The institutional electronic database was searched for patients with primary pulmonary or tracheobronchial adenoid cystic carcinoma (ACC) or mucoepidermoid carcinoma (MEC). Histopathological diagnoses were verified from the pathology records and the available pathological material. Age, sex, and smoking history were recorded as demographic variables. Clinical and radiological variables included tumor localization, laterality, and tumor size. Tumor localization was classified as tracheal, right lung, or left lung. Tumor size was recorded in centimeters.

Patients with these centrally located tumors generally present with respiratory symptoms such as dyspnea or exertional dyspnea. Thoracic computed tomography performed based on clinical suspicion reveals the presence of a mass or lesion. Owing to the central location of these tumors, bronchoscopy is performed for diagnostic evaluation, and histopathological diagnosis is established by bronchoscopic biopsy. Therefore, when significant airway obstruction is identified during bronchoscopy, endobronchial treatment may be performed in collaboration with the interventional pulmonology team, or tumor debulking may be undertaken to restore airway patency and reduce the tumor burden. In our series, endobronchial treatment consisted of cryorecanalization (cryo-extraction) of the endobronchial tumor. The tumor tissue was removed using a cryoprobe, and any residual tumor tissue was subsequently coagulated using argon plasma coagulation (APC). The definitive treatment strategy is subsequently determined by a multidisciplinary tumor board based on the histopathological evaluation.

**Figure 2:**
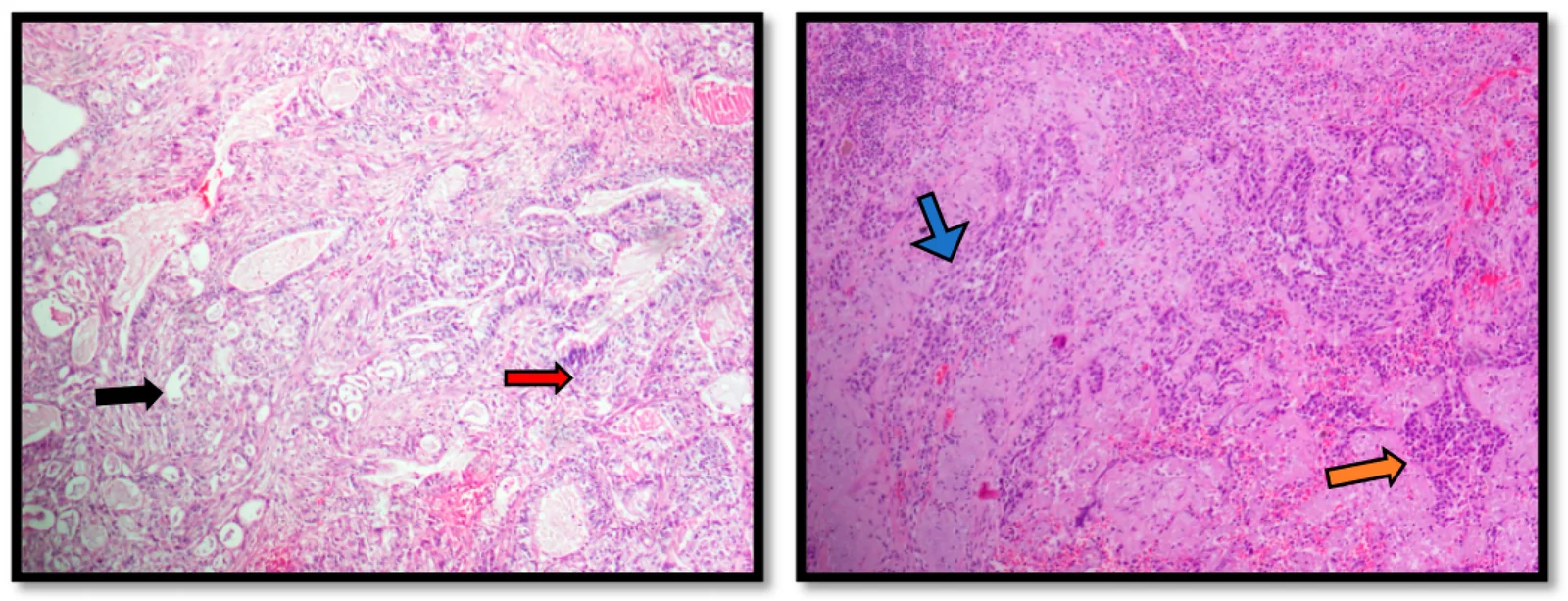
Low grade mucoepidermoid carcinoma and high grade mucoepidermoid carcinoma. Black arrow: mucin-containing glandular structures; red arrow: intermediate cells; Blue arrow: intermediate cells; orange arrow: squamous cells.

**Figure 3:**
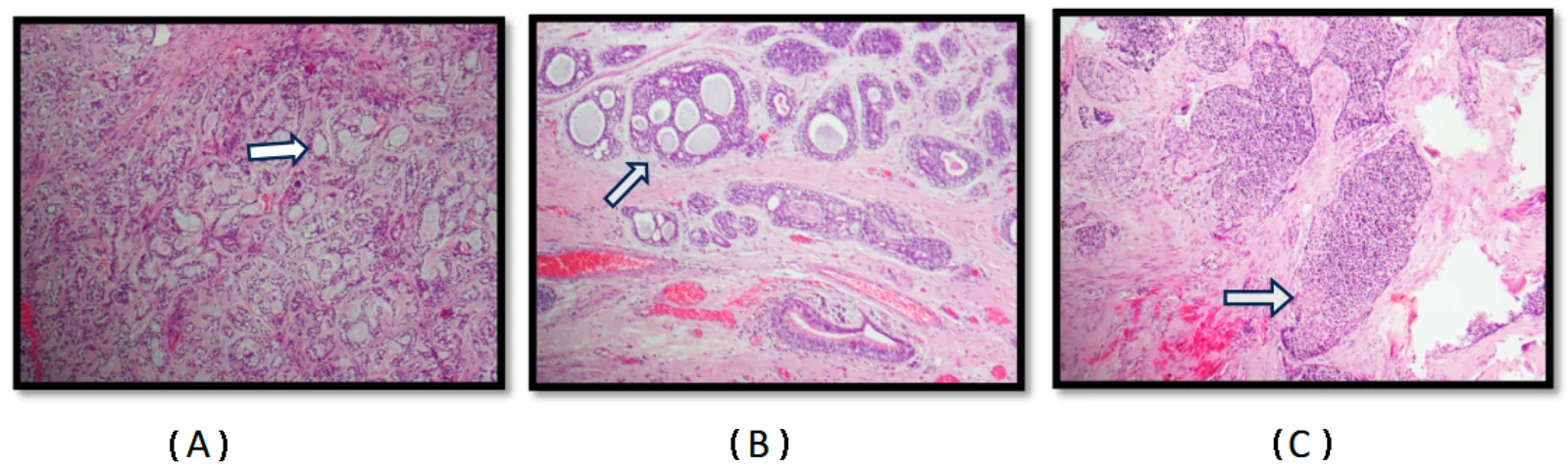
Representative histopathological features of primary pulmonary adenoid cystic carcinoma. (**A**) Predominantly tubular pattern showing small duct-like/tubular structures. (**B**) Predominantly cribriform pattern characterized by tumor nests with multiple pseudocystic spaces. (**C**) Predominantly solid pattern showing compact tumor nests with reduced tubular or cribriform architecture. Hematoxylin and eosin staining.

Cases were classified into two main histological groups: MEC (Figure 2) and ACC (Figure 3). Immunohistochemical assessment; an appropriate positive control was included in each immunohistochemical staining run. Immunohistochemical findings were interpreted together with the histomorphology. Diagnosis of MEC: histochemical mucin stains and diastase–periodic acid–Schiff (d-PAS) were used to demonstrate intracytoplasmic mucin in morphologically suspected mucous cells. These stains were interpreted as supportive findings and not as stand-alone diagnostic criteria. The immunohistochemical panel included CK7, p63, p40, and CK5/6, interpreted together with the histological findings. ACC was diagnosed by its characteristic biphasic epithelial and myoepithelial/basaloid differentiation and cribriform, tubular, and/or solid architecture. Cribriform spaces containing basement membrane-like hyaline material were considered characteristic supporting features. The immunohistochemical panel included CK7, p63, p40, and S100, interpreted in the context of the morphology.

Tumor T and N stages were assigned according to the 9th edition of the TNM staging system, using available clinical, radiological, and pathological data. Pleural effusion was defined as radiologically detected pleural fluid. No histopathological pleural invasion was identified in these patients, and the effusions were not considered malignant pleural effusions.

In patients undergoing tracheal or airway resection and reconstruction, bronchoscopic evaluation was routinely performed during the early postoperative period, particularly within the first postoperative week and before hospital discharge. This examination was used to assess anastomotic integrity and healing and to exclude early anastomosis-related complications before safe discharge. During long-term follow-up, thoracic CT was the primary surveillance modality. Bronchoscopy was subsequently repeated when clinically indicated, particularly in patients who developed new respiratory symptoms or when local recurrence was suspected on imaging. The presence of distant metastasis was assessed based on clinical follow-up, radiological examinations, and patient records.

While the data were analyzed retrospectively through patient files, there was no missing data from the patients in the study. Statistical analyses were performed using IBM SPSS Statistics for Windows, version 26. Continuous variables were expressed as mean ± standard deviation, while categorical variables were presented as frequencies and percentages. Comparisons of continuous variables between the ACC and MEC groups were performed using the independent-samples *t*-test or Mann–Whitney U test, as appropriate according to data distribution. Categorical variables were compared using the chi-square test or Fisher’s exact test, as appropriate. Overall survival (OS) and disease-free survival (DFS) were analyzed using the Kaplan–Meier method. Survival curves were compared between groups using the log-rank test. All statistical tests were two-sided, and a *p* value < 0.05 was considered statistically significant.

## 3. Results

Baseline clinicopathological characteristics of the cohort according to histologic subtype are summarized in Table 1. The mean age of the entire cohort was 53.3 ± 14.8 years. Overall, 39 patients (66.1%) were male and 20 (33.9%) were female. No statistically significant difference was observed between the ACC and MEC groups in terms of sex distribution (*p* = 0.162). A history of smoking was present in 27 patients (45.8%) in the overall cohort. Smoking was significantly more frequent in the MEC group than in the ACC group (63.6% vs. 35.1%; *p* = 0.034).

Tumor localization and staging characteristics are also presented in Table 1. With regard to tumor localization, 26 cases (44.1%) were located in the trachea, 18 (30.5%) in the right lung, and 15 (25.4%) in the left lung. Tracheal localization was markedly more frequent among ACC cases (62.2%), whereas right- and left-lung localization predominated in MEC cases. A statistically significant difference was observed between the two histologic subtypes in terms of tumor localization (*p* < 0.001). The mean tumor diameter and the distributions of T stage and N stage did not differ significantly between the ACC and MEC groups (*p* = 0.144, *p* = 0.832, and *p* = 0.088, respectively).

The distribution of histopathological parameters according to histologic subtype is shown in Table 1. No statistically significant differences were observed between ACC and MEC groups in terms of lymphovascular invasion (*p* = 0.795), perineural invasion (*p* = 0.162), necrosis (*p* = 0.183), STAS (*p* = 1.000), pleural invasion (*p* = 0.715), or pleural effusion (*p* = 0.896). However, perineural invasion was numerically more frequent in the ACC group than in the MEC group (40.5% vs. 22.7%).

### 3.1. Treatment Modalities and Surgical Margin Status

Treatment modalities and surgical margin status are summarized in Table 1. In the overall cohort, the most common treatment modality was endobronchial treatment (*n* = 23, 39.0%). This was followed by lobectomy/bilobectomy (*n* = 12, 20.3%), tracheal resection (*n* = 10, 16.9%) and pneumonectomy (*n* = 5, 8.5%). The distribution of treatment modalities differed significantly between ACC and MEC groups (*p* = 0.014). Endobronchial treatment and tracheal resection were more commonly performed in the ACC group, whereas lobectomy/bilobectomy was more prominent in the MEC group.

Surgical margin evaluation revealed negative margins in 44 cases (74.6%) and positive margins in 15 cases (25.4%). Surgical margin positivity was significantly more frequent in the ACC group than in the MEC group (35.1% vs. 9.1%; *p* = 0.019).

### 3.2. Recurrence/Metastasis Findings

The characteristics of patients who developed recurrence or metastasis during follow-up are summarized in Table 2. During follow-up, recurrence or metastasis developed in a total of 13 patients (22.0%). The recurrence/metastasis rate was significantly higher in the ACC group than in the MEC group (32.4% vs. 4.5%; *p* = 0.020). Among patients who developed recurrence or metastasis, the most common sites of involvement were the ipsilateral lung and trachea; bilateral lung, brain, and liver metastases were observed in fewer cases.

### 3.3. Survival Outcomes

**Figure 4:**
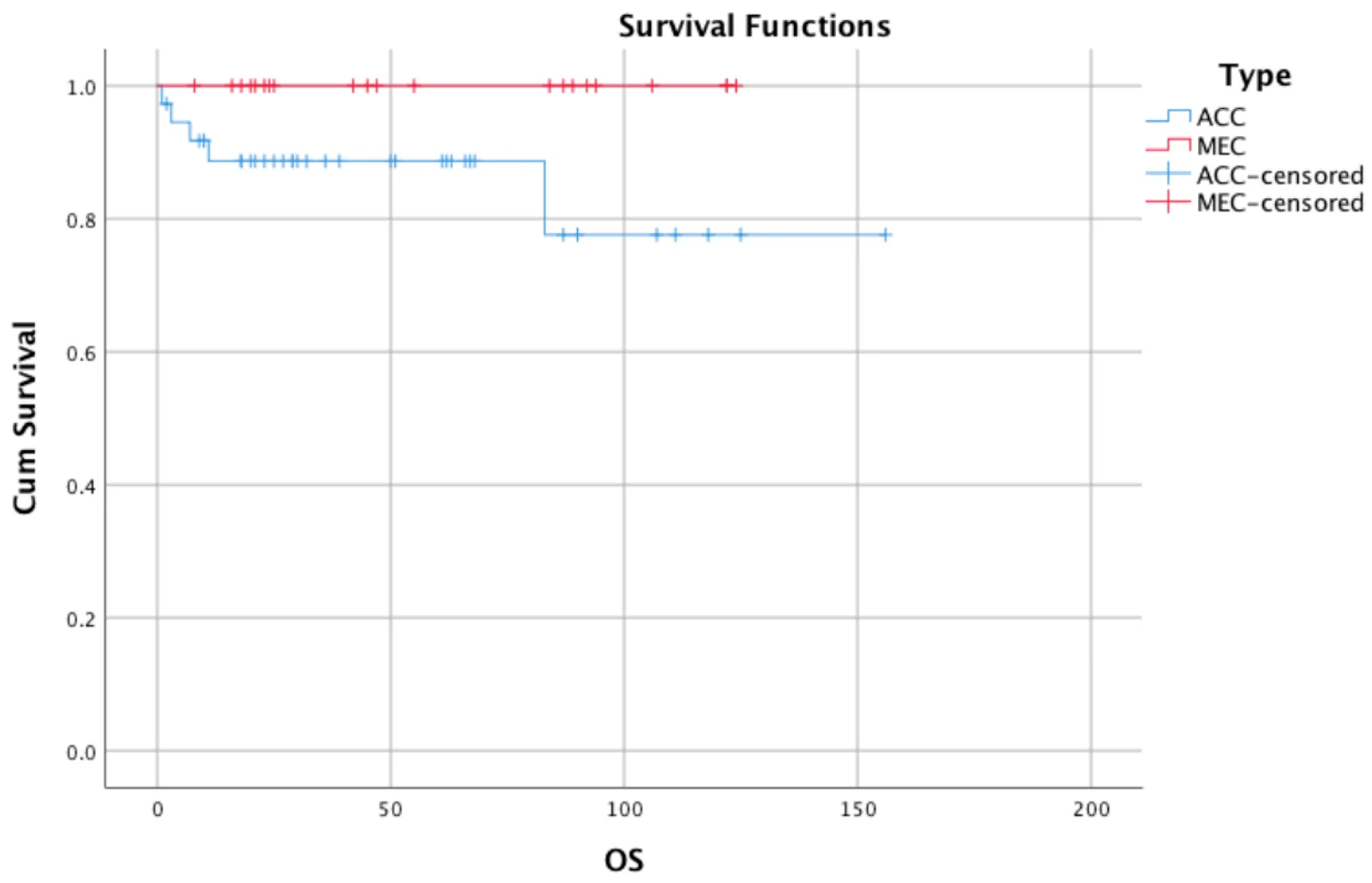
Kaplan–Meier Overall Survival Curves According to Histological Subtype (Adenoid Cystic Carcinoma vs. Mucoepidermoid Carcinoma).

Survival outcomes according to histologic subtype are summarized in Table 3 and illustrated by Kaplan–Meier curves in Figure 4. In the overall survival analysis, the 3-year and 5-year overall survival rates in the ACC group were both 88.7%, while in the MEC group, the 3-year and 5-year overall survival rates were 100%. Overall survival did not differ significantly between the ACC and MEC groups (*p* = 0.530).

**Figure 5:**
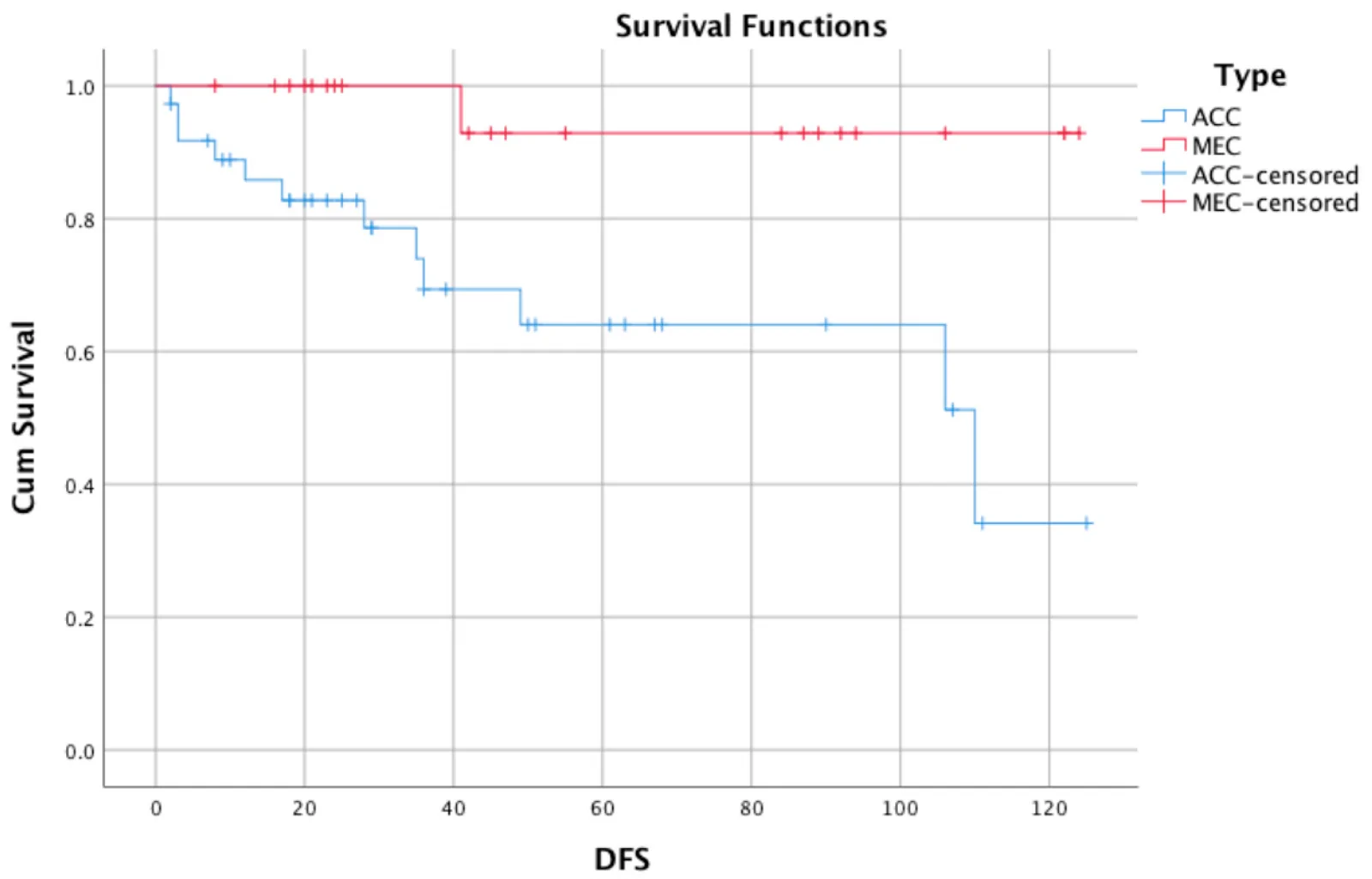
Kaplan–Meier Disease-Free Survival Curves According to Histological Subtype (Adenoid Cystic Carcinoma vs. Mucoepidermoid Carcinoma).

In the disease-free survival analysis, the 2-year and 5-year disease-free survival rates in the ACC group were 82.7% and 64.0%, respectively. In the MEC group, the 2-year and 5-year disease-free survival rates were 92.9%. Disease-free survival was significantly lower in the ACC group than in the MEC group (*p* = 0.009) (Figure 5).

Subgroup analyses evaluating prognostic factors for survival. When lymphovascular invasion, perineural invasion, necrosis, and STAS were evaluated in relation to disease-free survival, no statistically significant associations were detected (*p* = 0.102, *p* = 0.549, *p* = 0.714, and *p* = 0.785, respectively). Similarly, no significant difference was observed between tumor localization and disease-free survival (*p* = 0.346).

Surgical margin status was significantly associated with disease-free survival. In cases with positive surgical margins, the 2-year and 5-year disease-free survival rates were 70% and 59.1%, respectively, whereas the corresponding rates in cases with negative surgical margins were 95.3% and 80%. Disease-free survival was significantly lower in patients with positive surgical margins (*p* = 0.036).

ACC cases were further evaluated according to their dominant histopathological patterns. Representative histopathological images of tubular, cribriform, and solid patterns of adenoid cystic carcinoma are shown in Figure 3A–C. The 5-year overall survival rate was 86.2% in cases with a predominantly tubular pattern, 80% in cases with a predominantly cribriform pattern, and 50% in cases with a predominantly solid pattern. A statistically significant difference in overall survival was observed among dominant histopathological patterns (*p* = 0.034). However, no significant difference was detected in disease-free survival according to dominant pattern (*p* = 0.281).

## 4. Discussion

Primary pulmonary adenoid cystic carcinoma (ACC) and mucoepidermoid carcinoma (MEC) are traditionally grouped as salivary gland-type tumors; however, contemporary pathological understanding emphasizes that they represent biologically distinct neoplasms with different morphological, molecular, and clinical characteristics [7,8]. Recent studies similarly suggest that pulmonary salivary gland-type tumors constitute a heterogeneous group with subtype-specific biological behavior rather than a single clinicopathological entity [1,5,6,8]. In our cohort, these differences were reflected by more frequent tracheal involvement, a higher rate of positive surgical margins, increased recurrence or metastasis, and inferior disease-free survival in ACC compared with MEC.

From a modern pathological perspective, accurate classification of these rare tumors requires integration of morphology, immunohistochemistry, and, when necessary, characteristic molecular alterations [8]. ACC is characterized by biphasic differentiation and tubular, cribriform, and solid architectural patterns. These patterns may have biological and prognostic significance. In our cohort, a predominantly solid pattern was associated with poorer overall survival, supporting the concept that morphological heterogeneity in ACC may reflect differences in tumor biology. Molecularly, alterations involving MYB and MYBL1 are characteristic of ACC, while emerging evidence also suggests a role for NOTCH pathway alterations in aggressive disease [8]. Although molecular testing was not systematically performed in our cohort, future studies integrating molecular and morphological findings may provide a more refined understanding of prognosis.

The pathological growth characteristics of ACC may explain several of our clinical findings. ACC commonly arises in the central airways and is characterized by infiltrative growth, longitudinal submucosal extension, and a tendency toward perineural invasion [7,8]. These features may complicate the achievement of negative surgical margins, particularly in tumors involving the trachea and main bronchi. Consistent with this behavior, ACC in our cohort was more frequently located in the trachea and had a higher rate of positive surgical margins than MEC. This finding is also consistent with the pooled analysis by Garg et al., which demonstrated that ACC was more frequently located in the central airways than MEC [9]. Furthermore, positive surgical margins were associated with inferior disease-free survival in our cohort.

ACC is often considered a relatively slow-growing tumor; however, its indolent course should not be interpreted as the absence of biological aggressiveness. The tendency toward late local recurrence and distant metastasis may allow prolonged overall survival despite recurrent disease [2,3,7]. In the pooled analysis by Garg et al., recurrence was also substantially more frequent in ACC than in MEC, supporting the more pronounced tendency of ACC toward disease recurrence [9]. Similarly, in our cohort, ACC was associated with a higher rate of recurrence or metastasis and significantly inferior disease-free survival. However, overall survival did not differ significantly between ACC and MEC. This finding is in line with the population-based study by Qin et al., which similarly reported no significant difference in overall survival between patients with ACC and MEC despite differences in clinical characteristics between the two subtypes [10]. Taken together, these findings suggest that disease-free survival and recurrence patterns may provide complementary information beyond overall survival when evaluating the clinical burden of ACC.

In contrast, MEC showed a more favorable clinical profile in our cohort. However, pulmonary MEC should not be considered uniformly low-risk. Its biological behavior is influenced by histological grade, with high-grade tumors demonstrating greater cytological atypia, increased mitotic activity, necrosis, and a potentially more aggressive clinical course [8,11]. Molecular pathology has further contributed to the modern understanding of MEC, with MAML2 rearrangements, including CRTC1::MAML2 and CRTC3::MAML2 fusions, representing characteristic alterations that may assist in diagnosis in morphologically challenging cases [8,11]. Molecular testing was not routinely performed in our cohort, representing a limitation of the study and highlighting an important direction for future research.

The differences observed between ACC and MEC in our cohort extend beyond histological terminology and likely reflect their distinct underlying biology. ACC was more commonly associated with central airway involvement, difficulty in achieving negative surgical margins, recurrence or metastasis, and inferior disease-free survival, whereas MEC generally demonstrated a more favorable clinical course. Previous pooled and population-based studies have also demonstrated that clinical outcomes in pulmonary salivary gland-type tumors are influenced by factors such as tumor location, tumor size, metastatic status, histological grade, and surgical treatment [9,10]. These findings support a subtype-specific approach to these tumors and suggest that pathological subtype and growth characteristics should be considered together with anatomical location during surgical planning and postoperative follow-up [7,8].

Surgery should be considered as the primary treatment when complete resection is feasible, particularly given the central airway location of these tumors. In patients who are not initially suitable for complete resection, especially those with significant airway obstruction, endobronchial treatment can be used to restore airway patency. Neoadjuvant chemotherapy may then be considered in selected patients, followed by reassessment for surgical resection. However, evidence regarding neoadjuvant treatment in pulmonary ACC and MEC is still limited. The role of immunotherapy is also unclear, but with further molecular studies, immunotherapy may have a greater role in the treatment of these tumors in the future.

Our study has several limitations. Its retrospective and single-center design limits the generalizability of the findings, and the small number of patients and survival events reduced statistical power. Follow-up duration was also heterogeneous. In addition, molecular testing for characteristic alterations such as MYB/MYBL1 in ACC and MAML2 rearrangements in MEC was not systematically performed, preventing correlation between molecular findings, morphology, and clinical outcomes. Nevertheless, the present study provides a clinicopathological and surgical comparison of the two most common primary pulmonary salivary gland-type malignancies and highlights the importance of interpreting clinical outcomes within the context of modern pathological understanding.

## Figures and Tables

**Table 1:** Demographic and Clinicopathological Characteristics of the Patients According to Histological Subtype.

Variables	Total (*n* = 59)	Adenoid Cystic Carcinoma (*n* = 37)	Mucoepidermoid Carcinoma (*n* = 22)	*p* Value
Mean age ± SD (years)	53.3 ± 14.8	54.4 ± 13.3	51.5 ± 17.3	0.882
Gender				0.162
Male	39 (66.1)	22 (59.5)	17 (77.3)	
Female	20 (33.9)	15 (40.5)	5 (22.7)	
Smoking history	27 (45.8)	13 (35.1)	14 (63.6)	0.034
Tumor location				<0.001
Trachea	26 (44.1)	23 (62.2)	3 (13.6)	
Right	18 (30.5)	8 (21.6)	10 (45.5)	
Left	15 (25.4)	6 (16.2)	9 (40.9)	
Mean tumor size ± SD (cm)	2.6 ± 1.8	2.8 ± 1.8	2.3 ± 1.9	0.144
T stage				0.832
T1	42 (71.2)	25 (67.6)	17 (77.3)	
T2	10 (16.9)	7 (18.9)	3 (13.6)	
T3	5 (8.5)	3 (8.1)	2 (9.1)	
T4	2 (3.4)	2 (5.4)	--	
N stage				0.088
N0	51 (86.4)	30 (81.1)	21 (95.5)	
N1	6 (10.2)	6 (16.2)	--	
N2	2 (3.4)	1 (2.7)	1 (4.5)	
Low grade	15 (68.2)	--	15 (68.2)	--
High grade	7 (31.8)	--	7 (31.8)	
Cribriform pattern	18 (48.6)	18 (48.6)	--	--
Tubular pattern	15 (40.5)	15 (40.5)	--	
Solid pattern	4 (10.8)	4 (10.8)	--	
Lymphovascular invasion	20 (33.9)	13 (35.1)	7 (31.8)	0.795
Perineural invasion	20 (33.9)	15 (40.5)	5 (22.7)	0.162
Tumor necrosis	6 (10.2)	2 (5.4)	4 (18.2)	0.183
STAS	2 (3.4)	1 (2.7)	1 (4.5)	1
Pleural invasion	9 (15.3)	5 (13.5)	4 (18.2)	0.715
Pleural effusion	5 (8.5)	3 (8.1)	2 (9.1)	0.896
Treatment modality				0.014
Endobronchial treatment	23 (39)	18 (48.6)	5 (22.7)	
Trachea resection	11 (18.6)	8 (21.6)	3 (13.6)	
Carinal sleeve resection	2 (3.4)	2 (5.4)	--	
Pneumonectomy	5 (8.5)	4 (10.8)	1 (4.5)	
Sleeve lobectomy	4 (6.8)	1 (2.7)	3 (13.6)	
Lobectomy/Bilobectomy	12 (20.3)	3 (8.1)	9 (40.9)	
Wedge resection	2 (3.4)	1 (2.7)	1 (4.5)	
Surgical margin				0.019
Negative	44 (74.6)	24 (64.9)	20 (90.9)	
Positive	15 (25.4)	13 (35.1)	2 (9.1)	
Recurrence/Metastasis	13 (22)	12 (32.4)	1 (4.5)	0.020

**Table 2:** Clinicopathological Characteristics of Patients Who Developed Recurrence or Metastasis.

No	Age	Gender	Histological Subtype	Recurrence/Metastasis	Vital Status
1.	48	F	Adenoid cystic carcinoma	Trachea	Dead
2.	47	M	Adenoid cystic carcinoma	Trachea	Dead
3.	51	M	Adenoid cystic carcinoma	Trachea	Alive
4.	55	F	Adenoid cystic carcinoma	Ipsilateral lung	Alive
5.	60	M	Adenoid cystic carcinoma	Trachea	Alive
6.	37	F	Adenoid cystic carcinoma	Ipsilateral lung	Alive
7.	59	M	Adenoid cystic carcinoma	Ipsilateral lung	Dead
8.	36	M	Adenoid cystic carcinoma	Bilateral lung	Alive
9.	62	M	Adenoid cystic carcinoma	Ipsilateral lung	Dead
10.	68	M	Adenoid cystic carcinoma	Brain	Alive
11.	54	M	Adenoid cystic carcinoma	Ipsilateral lung	Alive
12.	42	M	Adenoid cystic carcinoma	Liver	Alive
13.	37	M	Adenoid cystic carcinoma	Ipsilateral lung	Alive

Recurrences involving the ipsilateral lung and trachea were classified as local recurrences, whereas lesions involving the bilateral lungs, liver, and brain were considered distant metastases.

**Table 3:** Overall Survival and Disease-Free Survival According to Histological Subtype.

	Overall Survival (%)			Disease-Free Survival (%)		
Subtype	3-Year	5-Year	*p* Value	2-Year	5-Year	*p* Value
ACC	88.7	88.7	0.530	82.7	64	0.009
MEC	100	100		92.9	92.9	

## Data Availability

The data supporting the findings of this study are available from the corresponding author upon reasonable request.

## References

[1] Zhang Y., Liu X., Gu Y., Zhang S. (2023). Clinical, laboratory, pathological, and radiological characteristics and prognosis of patients with pulmonary salivary gland-type tumors. J. Cancer Res. Clin. Oncol..

[2] Kumar V., Soni P., Garg M., Goyal A., Meghal T., Kamholz S., Chandra A.B. (2018). A Comparative Study of Primary Adenoid Cystic and Mucoepidermoid Carcinoma of Lung. Front. Oncol..

[3] Resio B.J., Chiu A.S., Hoag J., Dhanasopon A.P., Blasberg J.D., Boffa D.J. (2018). Primary salivary type lung cancers in the National Cancer Database. Ann. Thorac. Surg..

[4] Bchir A., Ahmed S.B., Mokni M. (2022). Primary malignant salivary gland-type tumors of the lung: Clinico-pathological study of 10 cases. Pan Afr. Med. J..

[5] Batra N., Mishra P., Pai T., Jiwnani S., Karimundackal G., Tiwari V., Purandare N., Janu A., Noronha V., Joshi A. (2024). SALTT study: A retrospective analysis of 111 SAlivary gland tumors of Lung and Tracheobronchial Tree. Ann. Diagn. Pathol..

[6] Deb P.Q., Suster D.I. (2025). Outcome of Primary Lung Salivary Gland-Type Carcinoma: A Population-Based Study. Int. J. Surg. Pathol..

[7] Horio Y., Kuroda H., Masago K., Matsushita H., Sasaki E., Fujiwara Y. (2024). Current diagnosis and treatment of salivary gland-type tumors of the lung. Jpn. J. Clin. Oncol..

[8] Naso J.R., Roden A.C. (2024). Recent developments in the pathology of primary pulmonary salivary gland-type tumours. Histopathology.

[9] Garg P.K., Sharma G., Rai S., Jakhetiya A. (2019). Primary salivary gland-type tumors of the lung: A systematic review and pooled analysis. Lung India.

[10] Qin B.D., Jiao X.D., Liu K., Wu Y., He X., Liu J., Zang Y.-S. (2018). Clinical, pathological and treatment factors associated with the survival of patients with primary pulmonary salivary gland-type tumors. Lung Cancer.

[11] Hu S., Gong J., Zhu X., Lu H. (2022). Pulmonary Salivary Gland Tumor, Mucoepidermoid Carcinoma: A Literature Review. J. Oncol..

